# Supplementary material for: A humanized knock-in Col6a1 mouse recapitulates a deep-intronic splice-activating variant
Source: bioRxiv. 2024 Mar 22:2024.03.21.581572. Preprint. [Version 1] doi: 10.1101/2024.03.21.581572 (PMC10996637; doi:10.1101/2024.03.21.581572)
Supplement: Supplement 1 [file media-1.pdf]

Supplementary figures

A humanized knock-in *Col6a1* mouse recapitulates a deep-intronic splice-activating variant

Véronique Bolduc[1*,#], Fady Guirguis[1*], Berit Lubben[1], Lindsey Trank[1], Sarah Silverstein[1], Astrid Brull[1], Matthew Nalls[1], Jun Cheng[2], Lisa Garrett[2], Carsten G. Bönnemann[1,#]

[1]Neuromuscular and Neurogenetic Disorders of Childhood Section, National Institute of Neurological Disorders and Stroke, National Institutes of Health, Bethesda, MD.

[2] NHGRI Transgenic and Gene Editing Core, National Human Genome Research Institute, National Institutes of Health, Bethesda, MD.

*Contributed equally to this work

[#]Co-corresponding author: Véronique Bolduc (Veronique.bolduc@nih.gov), Carsten G. Bönnemann (Carsten.bonnemann@nih.gov)

Supplementary Figure S1. Isoforms detection in quadriceps using long-read RNA sequencing.

(A) Custom builds for the *Mus musculus* (mm)/*Homo sapiens* (hs) *Col6a1* transcripts. The bottom transcript includes the 72-nt-long pseudoexon (PE). **(B-C)** Transcript isoforms in quadriceps from an 8-week-old *Col6a1*$^{h/h}$ mouse (B) and an 8-week-old *Col6a1*$^{h+189T/h+189T}$ mouse (C) were automatically detected by VIsoQLR from the BAM files. Values represent the percent of classified reads. Only isoforms with frequency greater than 3% are depicted.

Supplementary Figure S2. Grip strength of *Col6a1* h+189T mice.

Absolute forelimb grip strength force of 4-, 8-, and 20-week-old male (A) or female (B) mice. Each data point represents one individual mouse. Data are presented as mean ± SD (n=4-8 mice). Statistical analyses were performed with one-way ANOVA followed by Tukey's multiple comparisons test. *$p<0.05$; **$p<0.005$, ***$p<0.0005$; ****$p<0.0001$.

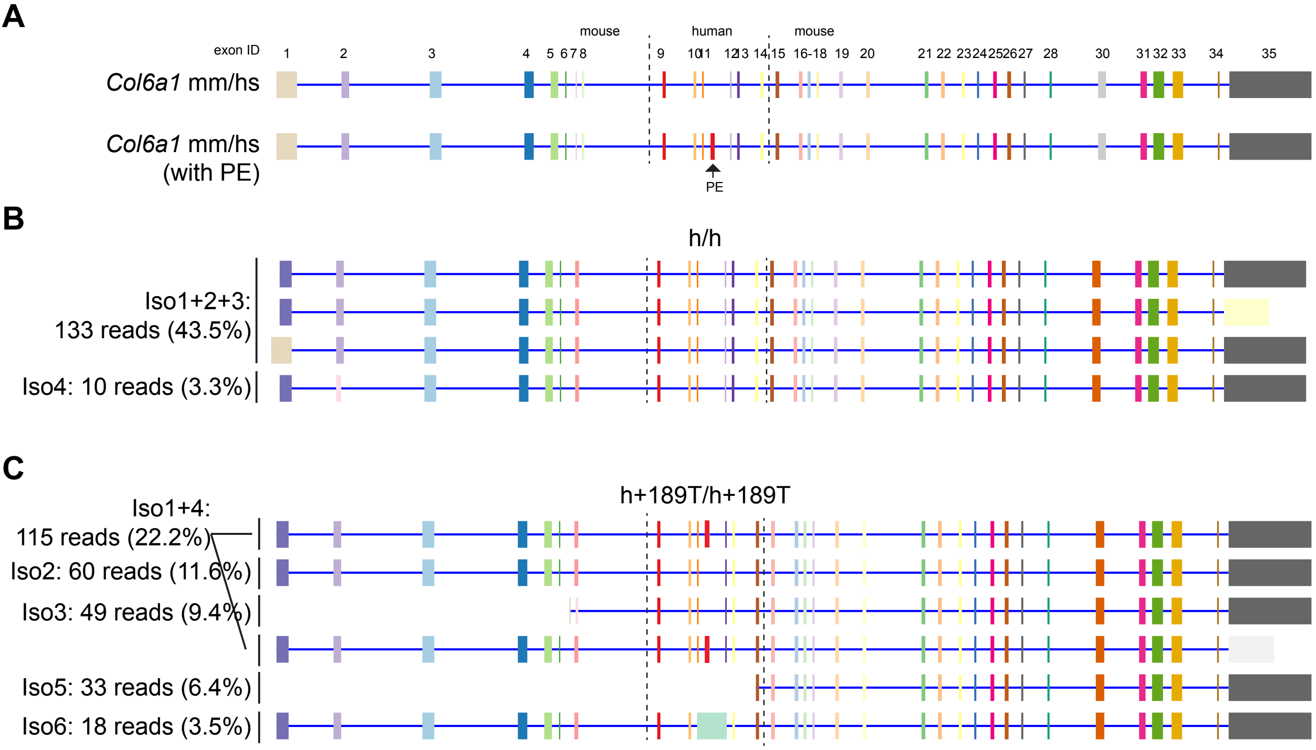

Supplementary Figure S1

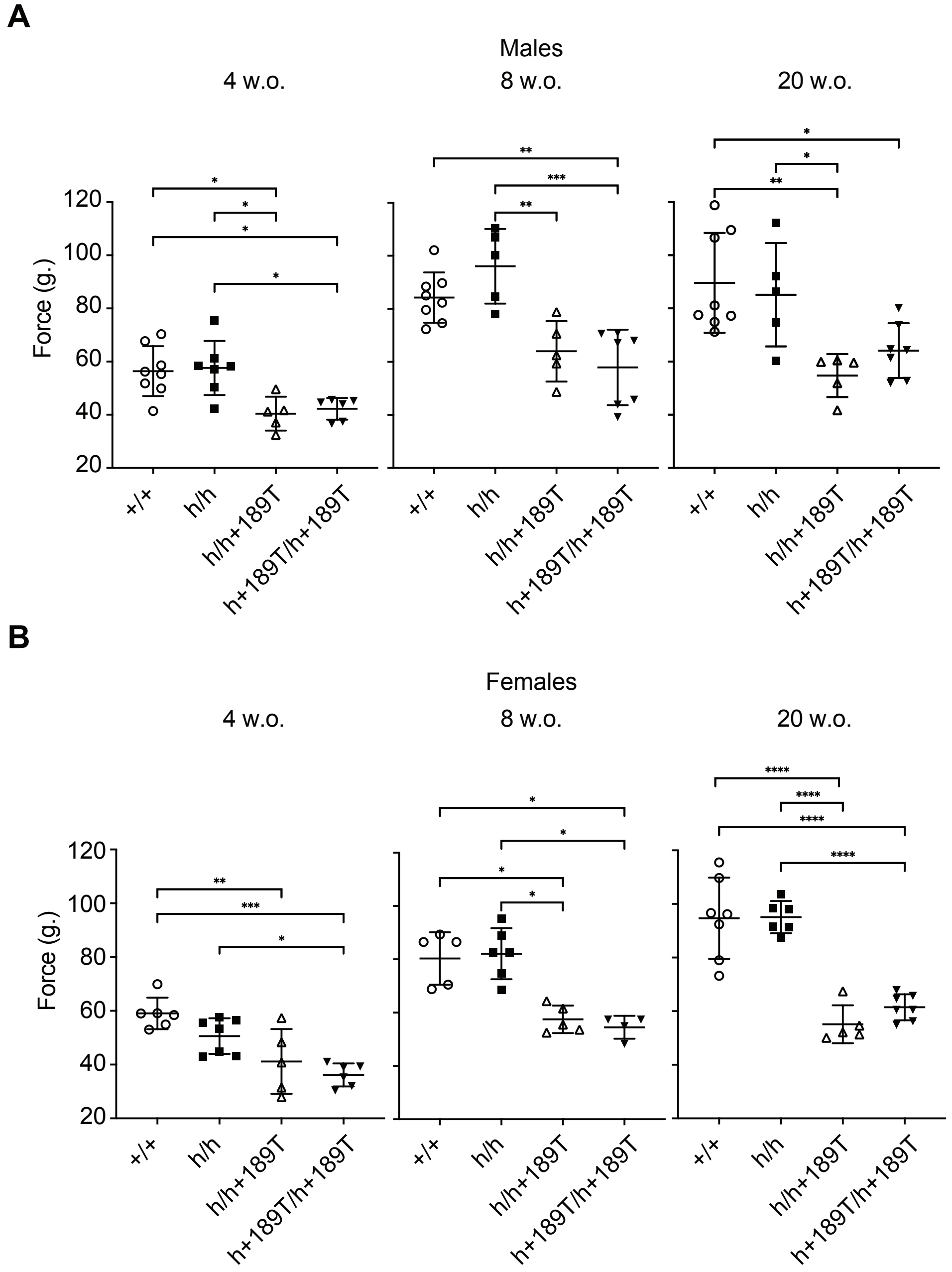

Supplementay Figure S2
